# Single‐cell and spatial omics unravel the spatiotemporal biology of tumour border invasion and haematogenous metastasis

**DOI:** 10.1002/ctm2.70036

**Published:** 2024-09-30

**Authors:** Xifu Cheng, Yuke Cao, Xiangyi Liu, Yuanheng Li, Qing Li, Dian Gao, Qiongfang Yu

**Affiliations:** ^1^ Department of Gastroenterology and Hepatology the Second Affiliated Hospital Jiangxi Medical College Nanchang University Nanchang China; ^2^ Department of Pathogen Biology and Immunology School of Basic Medical Sciences Jiangxi Medical College Nanchang University Nanchang China; ^3^ Queen Mary School Jiangxi Medical College Nanchang University Nanchang China; ^4^ Department of Oncology the Second Affiliated Hospital Jiangxi Medical College Nanchang University Nanchang China

**Keywords:** circulating tumour cell, single‐cell sequencing, spatial sequencing, tumour boundary, tumour cell behaviour, tumour microenvironment

## Abstract

Solid tumours exhibit a well‐defined architecture, comprising a differentiated core and a dynamic border that interfaces with the surrounding tissue. This border, characterised by distinct cellular morphology and molecular composition, serves as a critical determinant of the tumour's invasive behaviour. Notably, the invasive border of the primary tumour represents the principal site for intravasation of metastatic cells. These cells, known as circulating tumour cells (CTCs), function as ‘seeds’ for distant dissemination and display remarkable heterogeneity. Advancements in spatial sequencing technology are progressively unveiling the spatial biological features of tumours. However, systematic investigations specifically targeting the characteristics of the tumour border remain scarce. In this comprehensive review, we illuminate key biological insights along the tumour body‐border‐haematogenous metastasis axis over the past five years. We delineate the distinctive landscape of tumour invasion boundaries and delve into the intricate heterogeneity and phenotype of CTCs, which orchestrate haematogenous metastasis. These insights have the potential to explain the basis of tumour invasion and distant metastasis, offering new perspectives for the development of more complex and precise clinical interventions and treatments.

## Graphical abstract

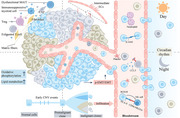

## 1 INTRODUCTION

Solid tumours consist of a differentiated core and an invasive border that reflects the invasive behaviour of the tumour with a distinctive cellular morphology and molecular composition.^1^ Metastatic cells often break away from the infiltrative margin of the primary tumour and enter the peripheral blood to form circulating tumour cells (CTCs), which are the ‘seeds’ of distant dissemination and exhibit significant heterogeneity.^2^, ^3^

Previous studies in tumour biology have primarily concentrated on delineating the tumour microenvironment (TME).^4^, ^5^ However, due to limitations of previous sequencing methods, exploration of different spatial regions, including tumour boundaries, has been somewhat overlooked.^6^ The intricate spatial biology that characterises tumours remains largely uncharted. In instances where metastases are initiated at the boundary, CTCs are accountable for seeding distant locales. Approximately a decade ago, one method for examining CTC heterogeneity was through liquid biopsy.^7^, ^8^ Nonetheless, this method furnishes only limited insights into CTC heterogeneity. The advantage of single‐cell and spatial omics technologies lies in their capacity to procure high‐resolution molecular information and precise spatial localisation, providing new fresh perspective for exploring the biological attributes of tumour boundaries and CTCs.^9^ With the aid of these tools, the panorama of tumour boundary invasion and metastasis mechanisms is gradually unfolding.

In this paper, we summarised the key spatiotemporal biological insights gleaned over the past five years regarding tumour border invasion and the mediated metastasis of CTCs. We provide an overview of the microenvironment components and tumour cell behaviour at the tumour boundary, as well as the biological characteristics of CTCs.

## 2 SINGLE‐CELL AND SPATIAL OMICS TECHNOLOGIES

The advantage of single‐cell sequencing technology lies in its high throughput and resolution. They are able to examine a wide range of molecular information within a single cell that has been assigned a specific identifier, including gene mutations, methylation sites, chromatin accessibility, RNA abundance and protein expression.^9^ Some technologies can even measure multiple molecular levels within a single cell.^36^ Single‐cell sequencing technology has been employed to identify rare cell subtypes, trace developmental lineages, and decipher intercellular signalling.^37^, ^38^, ^39^

**FIGURE 1:**
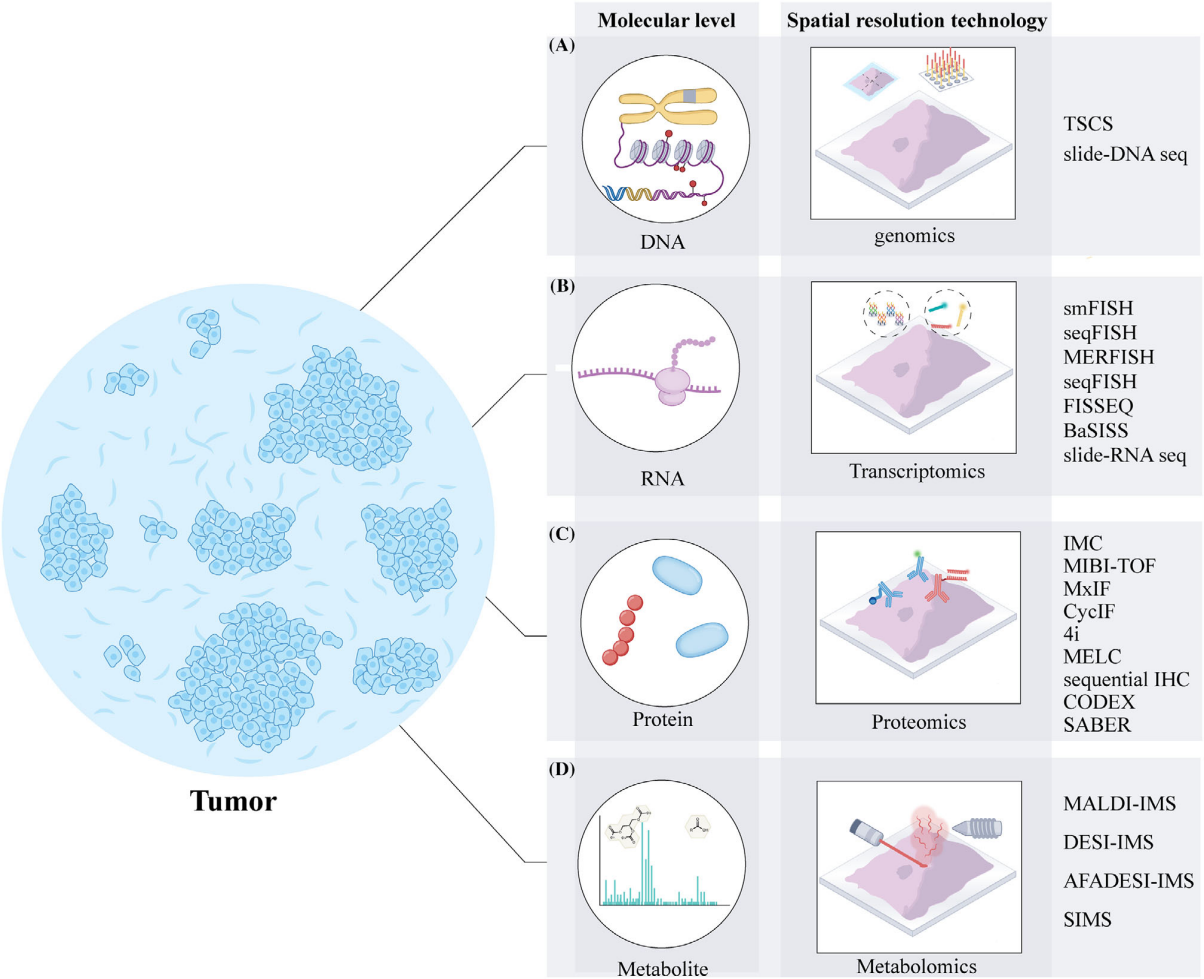
Spatial omics methods schematic. (A) DNA‐level spatial sequencing techniques predominantly employ micro‐dissection or bead‐based barcoding methods to analyse genomic information with spatial context. (B) RNA‐level spatial sequencing techniques commonly utilise spatial barcoding or in situ hybridisation imaging to assess transcriptomic profiles across different regions within a tissue. (C) Protein‐level spatial differential analysis primarily relies on mass spectrometry, complemented by fluorescence or colorimetric imaging, as well as oligonucleotide‐based fluorescence imaging or sequencing approaches for the detection and quantification of spatial protein expression. (D) Spatial metabolomics is founded on mass spectrometry imaging, enabling the spatial mapping and quantification of metabolites within tissue samples. TCSC, topographic single cell sequencing; smFISH, single‐molecule FISH; seqFISH, sequential FISH; MERFISH, multiplexed error‐robust FISH; BaSISS, base‐specific in situ sequencing; IMC, imaging mass cytometry; MIBI‐TOF, multiplexed ion beam imaging‐time of flight; MxIF, Multiplexed Immunofluorescence; 4i, iterative indirect immunofluorescence imaging; MELC, multi‐epitope‐ligand cartography; IHC, immunohistochemistry; CODEX, CO detection by indexing; SABER, Signal amplification by exchange reaction; MALDI‐IMS, matrix‐assisted laser desorption/ionisation IMS; DESI‐IMS, Desorption electrospray ionisation IMS; AFADESI‐ Desorption electrospray ionisation IMS, Airflow‐assisted desorption electrospray ionisation Desorption electrospray ionisation IMS; SIMS, secondary ion mass spectrometry.

The earliest attempts at high‐throughput spatial localisation sequencing of tissues can be traced back to laser capture microdissection (LCM) technology, though it has certain limitations in spatial resolution.^40^ Currently, spatial omics‐level sequencing technologies are still in development, with typical representatives including Visium, GeoMx DSP, and other tissue transcriptome capture methods based on spatial barcoding or in situ sequencing.^20^, ^41^ Protein imaging techniques with spatial resolution are mainly divided into methods based on mass spectrometry, fluorescence or chromogenic imaging, and oligonucleotide‐based fluorescent imaging or sequencing. Spatial metabolomics primarily integrates MSI (mass spectrometry imaging) and metabolomics techniques, leading to the development of various types of MSI (see Figure 1 and Table 1).

**TABLE 1:** Brief information on spatial technology.

Methods	Major molecular level	Principles	Typical spatial resolution	Refs
TSCS	DNA	LCM	Cellular	^10^
Slide‐DNA seq	DNA	Spatial index	10 µm	^11^
smFISH	RNA	In situ imaging	Subcellular	^12^
seqFISH	RNA	In situ hybridisation	Subcellular	^13^
MERFISH	RNA	In situ hybridisation	Subcellular	^14^
FISSEQ	RNA	In situ sequencing	Subcellular	^15^
BaSISS	DNA/RNA	In situ sequencing	Subcellular	^16^
Slide‐RNA seq	RNA	Spatial index	10 µm	^17^
Visium V1/V2	RNA	Spatial index	55 µm	^18^
Visium HD	RNA	Spatial index	2 µm	^19^
Xenium	RNA	in situ sequencing	Subcellular	^20^
GeoMx DSP	RNA/Protein	Spatial index	10 µm	^21^
Stereo‐seq	RNA	Spatial barcode	.22 µm	^22^
IMC	Protein	Mass spectrometry	1 µm	^23^
MIBI‐TOF	Protein	Mass spectrometry	.26 µm	^24^
MxIF	Protein	Fluorescence or chromogenic imaging	∼.2 µm	^25^
CycIF	Protein	Fluorescence or chromogenic imaging	∼.2 µm	^26^
4i	Protein	Fluorescence or chromogenic imaging	.165 µm	^27^
MELC	Protein	Fluorescence or chromogenic imaging	∼.2 µm	^28^
Sequential IHC	Protein	Fluorescence or chromogenic imaging	Subcellular	^29^
CODEX	Protein	Oligonucleotide‐based imaging	.25	^30^
SABER	Protein	Oligonucleotide‐based imaging	.32	^31^
MALDI‐IMS	Metabolite	Mass spectrometry imaging	5–200 µm	^32^
DESI‐IMS	Metabolite	Mass spectrometry imaging	100–500 µm	^33^
AFADESI‐IMS	Metabolite	Mass spectrometry imaging	40–100 µm	^34^
SIMS	Metabolite	Mass spectrometry imaging	.1–.5 µm	^35^

## 3 TUMOUR‐BORDER SUBCLONAL BEHAVIOUR

**FIGURE 2:**
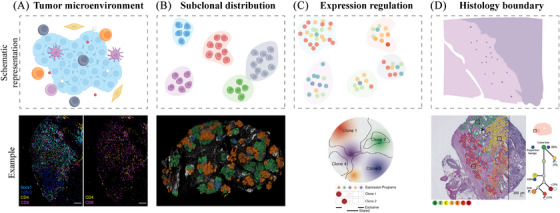
Subclonal behaviour of tumour bodies and boundaries. (A) The impact of subclonal distribution on interactions with the tumour microenvironment (TME). The representative images delineate the experimentally observed relationships between tumour clones and the TME. The blue Tgfbr2 knockout tumour clones exhibit a T‐cell excluded phenotype, whereas the cyan‐coloured subtype of Socs1 knockout has a stroma infiltrated by T cells. (B) The primary tumour's subclones are organised into clustered distributions within the tissue, constituting microanatomical structures with marginally overlapping boundaries. The depicted example pertains to the observation of dual subclonal distributions in a case of ductal breast carcinoma. (C) Variations in transcription patterns are observed among different subclones, with certain subclones exhibiting an enrichment of specific transcription factors. (D) A subset of subclones has disseminated into the peripheral stroma, transcending the histological confines. The accompanying examples graphically represent the distribution and evolutionary patterns of subclones within paracancerous tissue of prostate cancer. Note: All image materials have been duly authorised for use by Elsevier and Springer Nature. Part a from ref. (42). Elsevier. Part b from ref. (16). Springer Nature. Part c from ref. (43). Elsevier. Part d from ref. (44) Springer Nature.

Tumours consist of interlocking subclones and microenvironmental components.^45^ Tumour subclones usually show a mutually exclusive distribution due to differences in TME composition and ecological competition.^46^ At the same time, cancer cell populations also profoundly affect the spatial distribution of microenvironment components^47^ (Figure 2A). For example, the Perturb‐map technique for spatial CRISPR functional genomics was recently proposed. It enables the simultaneous knockout of multiple genes in a mouse lung cancer model to assess how each knockout affects tumour characteristics. The loss of TGF‐β signalling in Tgfbr2 knockout cancer cells leads to increased TGF‐β activation in the tumour stroma of mouse models, mediating fibroblast activation, which ultimately transforms the tumour into a fibromyxoid state and results in a T‐cell exclusion phenotype. In contrast, the loss of the Socs1 gene leads to increased T‐cell infiltration within the tumour.^42^

Owing to the stochastic nature of mutation accumulation during cancer cell division, cancerous regions commonly exhibit a blend of subclones.^48^ Spatial sequencing technology has unveiled that the survival and dissemination of cancer cells may not solely stem from genetic alterations but also from their spatial localisation. The clonal behaviour of tumours is equally influenced by the histological context.^49^ Numerous investigations have demonstrated that spatial constraints can dictate the trajectories of tumour evolution.^50^ Previous multiregional whole‐exome sequencing of tumour samples from 100 established lung cancer patients revealed that the majority of tumour regions (86%) were found to carry subclones from only a single branch of the phylogenetic tree.^51^ Spatial sequencing methodologies have illuminated that primary tumour subclones emerge and propagate in a more spatially organised fashion than previously understood (Figure 2B). For example, a new workflow called BaSISS (Base‐Specific In Situ Sequencing) has confirmed that each subclone of breast cancer occupies a discrete area within the tissue, thereby forming a microanatomical structure with slightly overlapping boundaries.^16^ Nonetheless, certain metastatic tumours manifest a more intricate subclonal landscape, characterised by monoclonal or mixed clonal metastases.^52^ Examination of various metastatic cancer types indicates that metastatic tumours tend to exhibit fewer intratumoural subclones compared to primary tumours.^53^, ^54^

RNA expression levels also mirror this heterogeneity.^55^, ^56^ Genomic alterations are associated with spatially distinct transcriptional profiles, indicative of diverse transcriptional landscapes resulting from genetic subcloning within tumours (Figure 2C). A recent study identified 57 genetic subclones in spatially resolved transcriptomic samples from 28 glioblastoma, with 26.32% of these subclones exhibiting a single transcriptional program.^43^ The similar phenomenon was observed in genetically engineered mouse models of lung adenocarcinoma. Through paired slide‐DNA‐seq and slide‐RNA‐seq, researchers spatially characterised two subclones exhibiting disparate transcriptional patterns.^11^

Distinguishing between premalignant clones and normal cells based on histological phenotype is not always feasible, as some early events in tumour evolution do not concurrently exhibit changes in cell morphology.^57^ In some instances, histopathological phenotypes may signify subclonal alterations.^58^, ^59^ But they are not always consistent with genomic behaviour,^60^ and premalignant clonal subsets may traverse histological boundaries (Figure 2D). A study observed a subclone in prostate cancer tissue with significant copy number variations (CNVs) on chromosomes 8 and 10, including amplification of the oncogene MYC and deletion of the tumour suppressor gene PTEN. This subclone is spatially located in the region of normal acinar cells.^44^ This observation may elucidate why certain patients with negative surgical margins still encounter recurrence and metastasis.^61^, ^62^

## 4 BOUNDARY INVASIVE PHENOTYPE: BORDER ECOLOGY AND TUMOUR CELL INVASION

The transition zone between tumour cells and adjacent normal tissue is critical for the local expansion and distant dissemination of malignant tumours, exhibiting complex microenvironmental changes and tumour cell characteristics.^63^, ^64^ While recent research has unveiled intriguing genomic insights about cells beyond histological boundaries, the current mainstream delineation remains based on histological phenotype. Consequently, when we refer to ‘boundaries’ in this context, we are referring to the interface defined histologically. In spatial and single‐cell omics studies, boundary regions differ from core regions in terms of TME composition, angiogenesis, metabolism, and tumour cell phenotypes.

### 4.1 Stromal and immune barriers: ecological safeguards at the tumour boundary

**FIGURE 3:**
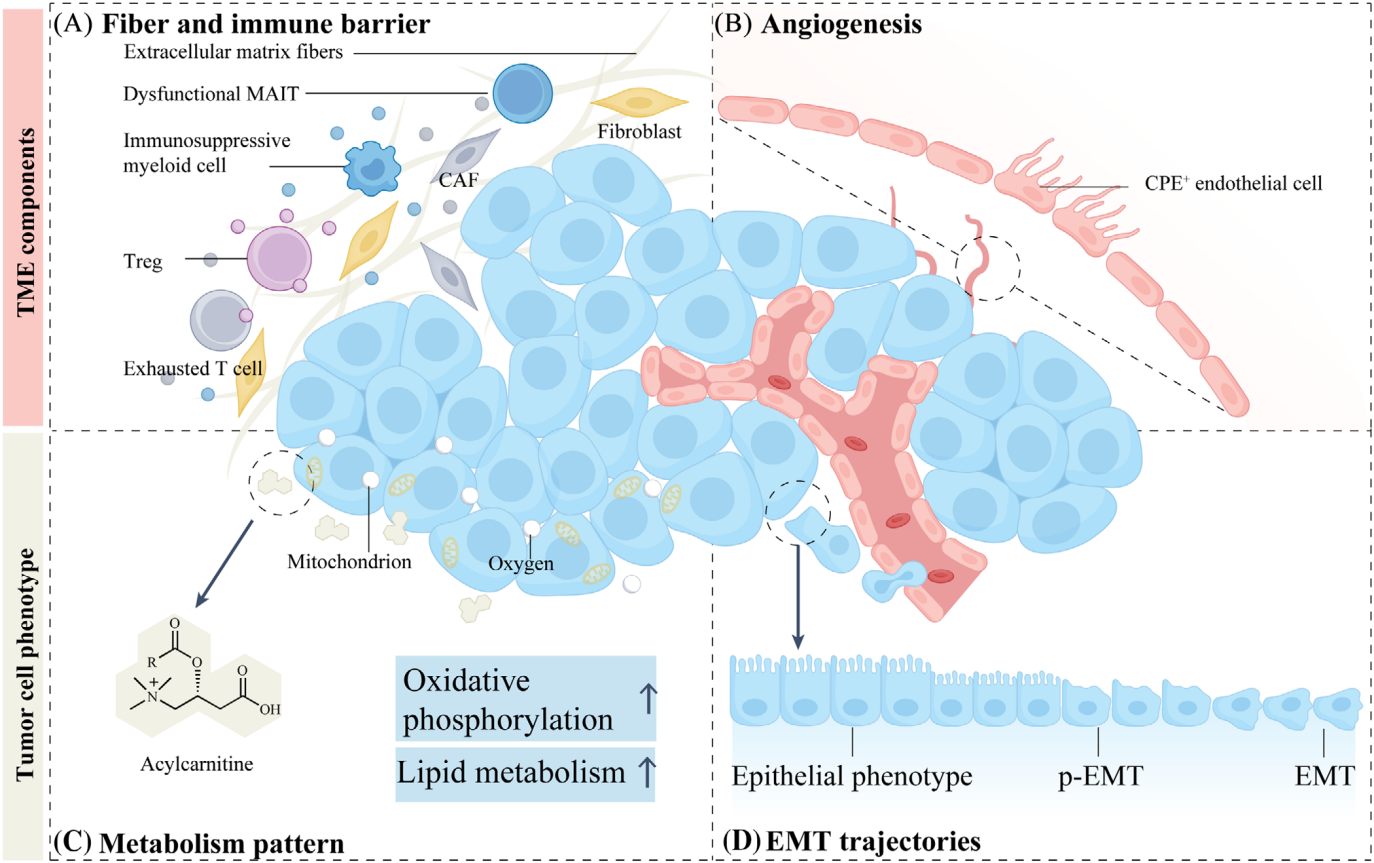
Components of the TME and tumour cell behaviour at the border zone. (A) The fibro‐immune barrier at the tumour periphery comprises noncellular fibres, cancer‐associated fibroblasts (CAFs), immunosuppressive myeloid cells, exhausted T cells, regulatory T cells (Tregs), and mucosal‐associated invariant T (MAIT) cells. (B) Along the tumour border, a robust angiogenic response occurs. Developing vascular endothelial cells exhibit an intermediate state between normal endothelial cells and tumour endothelial cells, including CPE^+^ endothelial cells. (C) Tumour cells at the border undergo metabolic reprogramming, characterised by elevated lipid metabolism and oxidative phosphorylation. (D) The evolutionary process of epithelial‐mesenchymal transition (EMT) involves three stages: complete epithelial type, partial EMT (p‐EMT), and complete EMT. Cancer cells at the border typically display p‐EMT and EMT phenotypes, detach from the primary tumour, and facilitate distant metastasis.

In the process of tumour invasion and immune evasion, the ecological barrier at the tumour boundary can hinder immune cell infiltration and function, playing a critical protective role as a barrier.^65^, ^66^ It is important to note that the observed stromal and immune cell barriers do not exist in isolation, but rather communicate with each other through known and unknown cytokines (Figure 3A). Additionally, this process may also involve the participation of cancer cells and endothelial cells.^67^ Some side evidence is that certain cytokines exhibiting spatial concentration gradient phenomena.^68^ For instance, in Göttingen minipigs spinal cord glioma models, elevated levels of TNF‐α, IL‐1β, and IL‐6 were discerned in the peritumoural region relative to the tumour core.^69^ Over the past five years, spatial ecological gradients at these critical boundaries have been meticulously explored using cutting‐edge single‐cell and spatial omics techniques (see Table 2).

**TABLE 2:** Key findings in tumour boundary.

Cancer type	Methodology	Boundary key findings	References
Brain cancer	LCM	Fibrous Collagens Expression: COL2A1, COL11A1, and COL11A2 exhibit elevated expression levels within the solid tumour region. Conversely, COL1A2 and COL3A1 are more abundant at the brain/tumour interface.	^70^
CRC, HCC	scRNA‐seq, Visium ST	The tumour border was significantly enriched in APSN^+^ fibroblasts and SPP1^+^ macrophages compared to other regions. Analysis of intercellular signalling communication established SPP1^+^ macrophages as the main senders of the SPP1 signalling pathway, APSN^+^ fibroblasts as the main secretors of collagen, and malignant cells as the main senders of the MIF signalling pathway.	^71^
Breast cancer	IMC	A multi‐layer spatial analysis of 693 breast tumours revealed a significant enrichment of PDPN‐expressing myofibroblasts at the border.	^72^
Pan‐cancer	InDrop, Visium ST	ECM‐CAF is enriched at tumour boundary. Notably, its level is significantly higher in non‐responders compared to responders. ECM‐CAF interacts with malignant cells, promoting tumour progression. Additionally, it regulates exhausted CD8^+^ T cells through immune checkpoint ligand receptors (e.g., LGALS9/TIM‐3), facilitating immune evasion.	^73^
Liver cancer	Visium ST, scRNA‐seq	SPP1 macrophages and CAFs co‐localise around tumours in ICB non‐responders to form a tumour immune barrier. Blockade of SPP1 or macrophage‐specific deletion of Spp1 resulted in enhanced efficacy of anti‐PD‐1 therapy in a mouse model of hepatocellular carcinoma, accompanied by a reduced proportion of CAF infiltration and an increased proportion of cytotoxic T‐cell infiltration.	^74^
Liver cancer	Visium ST	Bidirectional interactions between ligands and receptors at 100 µm‐wide cluster‐cluster boundaries help to maintain intratumoural architecture. An intact fibrous capsule, composed of fibroblasts and endothelial cells, can act as a barrier against immune cell infiltration.	^75^
HCC	Visium ST, scRNA‐seq	CSC and SPP1^+^ macrophages co‐localise at the border. SPP1 signalling is most active in cell‐to‐cell communication, including SPP1‐CD44 and SPP1‐ITGA/ITGB. CD8^+^ T cells are excluded from the tumour area, and HIF1A and HAVCR2 are highly expressed at the border of the co‐localise area.	^76^
GBM	scRNA‐seq	Many downregulated genes in tumour‐infiltrating peripheral CD8^+^ T cells are associated with classical IFN responses (IFI6, IFI27, MX1, STAT1, EPSTI1 PARP9, ISG15), cell proliferation (STMN1, CENPF, HELLS, NUSAP1, and DNPH1), and T cell costimulation (CD28, TMIGD2, TNFRSF4, CD27, and TNFRSF18).	^77^
TNBC	CODEX SPMIBI‐TOF	Two distinct TME themes emerge near the tumour‐immune boundary. In the first theme, immune cells co‐express high levels of indoleamine‐azole 2,3‐dioxygenase (IDO), programmed death ligand 1 (PDCD1), integrin αM (CD11b), and integrin αX (CD11c). The second theme is located closer to or directly at the tumour‐immune boundary itself. Immune cells in this theme also exhibit elevated expression of CD45 and FOXP3, indicative of immunosuppressive regulatory T cells.	^78^
Breast cancer	CODEX, Xenium ST, Visium ST	A decrease in tumour cell numbers is observed from the intratumoural region to the stromal region. Notably, Treg cells and plasma cells are uniquely enriched at the tumour border.	^79^
HCC	scRNA‐seq, CODEX	MAIT Cells and TAM Interaction: MAIT cells are enriched in the tumour edge region. CSF1R^+^PDCD1^+^ tumour‐associated macrophages (TAMs) exhibit strong signal communication with MAIT cells at the tumour edge. This interaction impairs MAIT cell function through direct contact and PDCD1‐dependent mechanisms.	^80^
HCC	CyTOF, 10x Genomics scRNA‐seq	Tumour‐Associated Double‐Positive T Cells: Tumour‐associated double‐positive T (DPT) cells, with immune activity and memory‐like phenotype, are enriched at the invasive front. These cells express PD‐1, HLA‐DR, ICOS, and CD45RO.	^81^
Primary Melanoma	CycIF, NanoString GeoMx DSP	PD1 CTLs and Immune Cells at the Border Zone: Frequent contacts occur between PD1‐expressing cytotoxic T lymphocytes (CTLs) invading the border zone and PDCD1‐expressing macrophages or dendritic cells. IDO1, indirectly activating Tregs and myeloid‐derived suppressor cells (MDSCs), is also enriched in this region.	^68^
CRC	Visium ST, scRNA‐seq	FAP fibroblasts in the border zone colocalise and interact with SPP1 macrophages. This interaction may be regulated by chemoattractant proteins, TGF‐β, and interleukin‐1. Notably, the high infiltration of these cells is associated with immunotherapy resistance.	^82^
CRC	Visium ST, scRNA‐seq	Colorectal cancer cells at the invasive front play a role in transforming macrophages into SPP1^+^ macrophages. This transformation occurs through the secretion of HLA‐G by the cancer cells. SPP1^+^ macrophages recruited by this process then recruit immune‐suppressive cells via cytokines and chemokines. Additionally, they reduce cytotoxicity through immune checkpoints.	^83^
Liver cancer	Stereo‐seq, scRNA‐seq	Immune checkpoint genes, including CTLA4, CD96, and TIGIT, are enriched in the first layer on the tumour side. Within the 250 µm wide region adjacent to the tumour border, the damaged area induces high expression of SAA1 and SAA2 by CXCL6. This expression pattern recruits macrophages and promotes their M2 polarisation.	^84^
Breast cancer lung metastases	NICHE‐seq	TREM2^+^ regulatory macrophages exhibit spatial differences. They express features related to ECM remodelling and lipid metabolism. These macrophages form inhibitory niches specifically at the invasive edge of metastases.	^85^
Tonsil cancer	NanoString GeoMx DSP	In the border zone, myeloid antigen‐presenting cells (APCs) expressing CD11c exhibit specific characteristics. They have elevated levels of immunosuppressive markers, such as B7‐H3 and Tim3. Interestingly, their surface protein HLA‐DR levels are decreased, impacting antigen recognition and presentation.	^86^

#### 4.1.1 Extracellular matrix and fibroblasts

Spatial omics studies have confirmed that the fibrous structure of the boundary zone affects the infiltration of T cells and myeloid cells in the tumour.^75^ Macroscopically, fibroblasts in the TME can be divided into two categories: normal fibroblasts and cancer‐associated fibroblasts (CAFs). Fibroblasts play an indispensable role in tumour immune desert and immune excluded phenotype due to their ability to synthesise and secrete collagen. Several single‐cell studies have pointed out the highly heterogeneous origins of CAF differentiation and their impact on immune cell function and the extracellular matrix.^87^, ^88^, ^89^ Recent spatial proteomics analysis showed that COL1A2 and COL3A1 were more abundant in the region where the brain and tumour interface.^70^ Significant enrichment of fibroblasts with APSN^+^, FAP^+^, and PDPN^+^ was observed in studies of hepatocellular carcinoma, colorectal carcinoma breast cancer, and was involved in limiting T cell infiltration.^71^, ^72^, ^82^ In addition, some specific CAFs are distributed at the borders of certain tumours, regulating immune cells with immunosuppressive phenotypes. For instance, ECM‐CAFs enriched at the tumour border act as a barrier to exclude immune cells. They also interact with malignant cells to promote tumour progression. Additionally, immune checkpoint ligand receptors (such as LGALS9/TIM‐3) regulate depleted CD8^+^ T cells to promote immune escape.^73^ Notably, this phenomenon extends beyond specific tumour types, as demonstrated in a liver cancer study where CAFs contribute to barrier formation, impacting the effectiveness of immunotherapy.^74^

#### 4.1.2 T lymphocytes

At the boundary interface, a subset of T cells exhibits dysfunction and exhaustion, culminating in compromised innate and specific anti‐tumour immunity. This boundary effect emerges as a primary driver of tumour expansion.^90^, ^91^ Recent spatial omics studies have unveiled the expression of several T cell exhaustion genes within this critical region, including PDCD1, LAG3, HAVCR2, 4‐1BB, and Tim‐3.^75^, ^76^ Simultaneously, classic interferon (IFN) response characteristics, cell proliferation, and T cell costimulation are downregulated.^77^ Treg enrichment at the tumour border is supported by the results of several studies on breast cancer.^78^, ^79^ Additionally, some other types of T cells have been found to become dysfunctional, such as MAIT.^80^ However, some studies have reported contrary results, indicating the functional and phenotypic diversity of T cells in the border region. For example, possible differentiation derived from single‐positive T‐cell tumour‐associated CD4/CD8 double‐positive T (DPT) cells recently identified at the tumour border exhibit synergistic expression of PD‐1, HLA‐DR, ICOS, and CD45RO. Upon stimulation these DPT cells produced high levels of IFN‐γ, TNF‐α and PD‐1 mediated antitumour responses.^81^ This new finding challenges the traditional developmental lineage in which DPT cells differentiate into single‐positive T cells after positive selection, highlighting the complexity of T cell‐related immunity at the tumour boundary.^92^ The balance between anti‐tumour and pro‐tumour capabilities of T cell populations may reflect certain boundary factors as to why there is no residue recurrence after early tumour treatment and unrestricted growth of late‐stage cancer.^93^, ^94^

#### 4.1.3 Myeloid cells

Emerging omics studies have further illuminated the existence of myeloid cell‐enriched tumour boundaries in specific malignancies.^68^ Extensive single‐cell sequencing studies have revealed the crucial role of myeloid cell subsets in immune crosstalk within the tumour microenvironment (TME) and their involvement in treatment resistance.^95^, ^96^, ^97^, ^98^ Ye and colleagues' spatial omics analysis has identified the enrichment of SPP1+ macrophages at the borders of colorectal and liver cancers, where they engage in complex crosstalk with adjacent ASPN^+^/FAP^+^ fibroblasts and tumour cells.^71^, ^82^ A similar study observed the colocalisation of cancer stem cells and SPP1^+^ macrophages in the hypoxic regions at the tumour boundary.^76^ Functionally, this subtype aligns with an M2 immunosuppressive phenotype and actively participates in angiogenesis.^99^, ^100^ Additionally, Ozato et al. have demonstrated that HLA‐G^+^ tumour cells infiltrating the colorectal cancer border orchestrate the transformation of neighbouring macrophages into SPP1^+^ macrophages, thereby shaping a distribution gradient.^83^ Notably, the pivotal ligand‐receptor signalling axis involves SPP1‐CD44 and SPP1‐ITGA/ITGB.^74^, ^76^ This boundary recruitment effect may also underlie the compromised invasion of the peripheral stroma. A study revealed that damaged hepatocytes located in the pre‐infiltration region of hepatocellular carcinoma secrete serum amyloid A1 and A2, which attract FPR1^+^ and TLR2^+^ macrophages and promote their polarisation into the M2 phenotype.^84^ Beyond SPP1^+^ macrophages, other myeloid cell subtypes have emerged as critical players at the tumour border. PDCD1^+^ and TREM2^+^ macrophages, identified through spatial omics analyses, occupy this dynamic niche.^68^, ^81^, ^85^ Notably, TREM2 expression predominantly resides within TAMs within the TME.^101^, ^102^, ^103^ Functionally, these subtypes have been definitively linked to the inhibition of T cell cytotoxicity and resistance to anti‐PD‐1 therapy in various mouse cancer models.^104^, ^105^, ^106^ These findings highlight the therapeutic potential of targeting specific myeloid cell subtypes to restore immune function at tumour boundaries and limit invasion.^107^, ^108^

### 4.2 Angiogenesis

Tumour growth necessitates the development of novel blood vessels from the pre‐existing vascular bed, ensuring an adequate supply of oxygen and nutrients.^109^ Spatial omics analyses have revealed a pronounced enrichment of angiogenic pathways in the border zone.^84^ Recent single‐cell analyses of tumour endothelial cells (TECs) have found that, compared to normal endothelial cells, TECs exhibit biological processes related to immune regulation and extracellular matrix organisation.^110^, ^111^, ^112^ Leveraging scRNA‐seq technology, researchers have identified endothelial cells with intermediate phenotypes.^111^, ^113^ Notably, Zhou et al. observed that CPE^+^ endothelial cells aggregate at the tumour‐peritumoural junction zone, displaying a differentiation state intermediate between liver sinusoidal endothelial cells (ECs) and TECs.^114^ CEBPD, ATF3, FOSB, and MYC were identified as potential regulatory transcription factors, similar to those reported in recently described fetal‐like endothelial cells.^113^ Differentiated intermediate state cells emphasise dynamic formation of bordering tumour vasculature (Figure 3B). However, our understanding of endothelial cell behaviour in the boundary zone remains limited, partly due to the small number of endothelial cells that can be captured within the tumour's components.^115^

### 4.3 Metabolism pattern

Typically, cancer cells enhance glucose and fatty acid uptake and reprogram metabolism to meet the demands of rapid proliferation.^116^, ^117^ The spatial distribution of nutrients restricts the metabolic phenotype, thereby influencing the biological behaviour of tumour cells.^118^ Spatial metabolomics techniques can directly map various metabolites from tissue sections, providing deep insights into the spatial complexity of tumour metabolism.^119^ Wang et al. used MALDI‐IMS to identify different tumour‐specific metabolic subtypes in 362 gastric cancer patients, revealing metabolic differences between tumours.^120^ Within tumours, metabolic differences can be characterised by a gradient between central and boundary regions. Notably, glucose and lipid metabolism stand out as prominent players, likely influenced by factors such as hypoxia and the heterogeneous distribution of nutrients.^121^ Banerjee et al., utilising DESI‐MSI made an intriguing observation: the glucose/citrate ratio significantly decreases at the boundary between malignant prostate tissue and adjacent prostate tissue.^122^ Interestingly, certain tumours exhibit robust lipid metabolism within the border zone. This metabolic shift may hold implications for cancer progression and metastasis, as increased fatty acid oxidation is often associated with these processes.^123^ For instance, 3D MALDI MSI revealed an enrichment of acylcarnitines throughout the invasive glioblastoma margin, underscoring their involvement in lipid metabolism.^124^ Furthermore, other types of spatial omics techniques have also revealed the metabolic characteristics of the boundary through enrichment of metabolic pathways, such as oxidative phosphorylation and lipid metabolism.^84^, ^125^ Certain tumour cell populations, characterised by oxidative phosphorylation metabolism, actively participate in metastasis at the border of breast cancer.^126^ These distinct metabolic profiles play a pivotal role in providing the necessary energy and raw materials to support invasion and metastatic processes at the tumour boundary (Figure 3C).

### 4.4 EMT spatiotemporal trajectories

One of the characteristics of tumour cell infiltration is dedifferentiation and disruption of normal tissue structure.^127^ Individual and collective cell migration primarily involves the epithelial‐mesenchymal transition (EMT) process.^128^ Notably, EMT dynamics are particularly pronounced at the invasion edges, unfolding in a phased manner (Figure 3D).^129^ Recent single‐cell and spatial omics studies have found that tumour cell subpopulations with an EMT phenotype are located at the boundaries of clear cell renal carcinoma, brain tumours, and liver cancer.^70^, ^84^, ^130^, ^131^ In colorectal cancer, malignant cell with enhanced EMT capabilities at the boundary are located at the endpoint of the evolutionary trajectory.^125^ It is worth noting that invasive front tumour budding potentially associated with EMT has been identified as a prognostic factor in cancers such as colorectal cancer,^132^, ^133^

Generally, EMT trajectories in cancer can be broadly categorised into three macroscopic states: full epithelial phenotype, mixed phenotype, and full mesenchymal phenotype. While many studies traditionally view EMT as a binary switch,^134^, ^135^, ^136^ this perspective overlooks the intrinsic and local microenvironmental influences that may evolve as EMT progresses along the continuum. Recent investigations have begun to revise this notion. Pioneering single‐cell analyses have identified seven EMT subtypes in primary tumours of the skin and breast, where cells exhibiting a hybrid EMT phenotype (co‐expressing both epithelial and mesenchymal markers) dominate in cancer metastasis.^137^ Consequently, cancer cells in the partial EMT (p‐EMT) state may exhibit greater invasiveness than those in the complete EMT state. For instance, a recent pan‐cancer single‐cell analysis reported a substantial frequency (39%) of mixed states, characterised by the simultaneous expression of epithelial and mesenchymal markers. This state is associated with heightened infiltration of CAFs, occasionally accompanied by enhanced hypoxia and stemness.^138^ Similar phenotypes are also observed at the tumour boundaries. Leveraging scRNA‐seq, the p‐EMT expression signature was delineated in subpopulations of tumour cells from head and neck squamous cell carcinoma and skull base chordoma, spatially localised at the leading edge of the primary tumour.^139^, ^140^ These studies mostly highlight the characteristic expression of the TGFBI gene induced by TGF‐β. The EMT effects at the boundary may be a response to peritumoural TME signals, involving key cytokines like TGF‐β and its activated Smad and non‐Smad signalling pathways, as well as the Wnt pathway.^141^, ^142^, ^143^, ^144^ For example, one study found that CAFs can stimulate TGF‐β expression in neighbouring tumour cells.^145^ These findings emphasise the dynamics and invasiveness of EMT at the tumour boundary.

## 5 HAEMATOGENOUS METASTASIS HETEROGENEITY: EXTRACELLULAR ENVIRONMENT AND CTC PHENOTYPES

Metastatic cells infiltrate the invasive margin of the primary tumour.^64^ Tumour cells can metastasise by directly invading blood vessels or the lymphatic system, or by spreading and implanting into adjacent tissues. A substantial body of literature supports the haematogenous route as the predominant mode of metastasis.^146^, ^147^ Single‐cell omics offers a high‐resolution perspective on the heterogeneity of circulating tumour cells (CTCs) implicated in haematogenous metastasis.

### 5.1 Spatial and temporal heterogeneity

The process of distant metastasis encompasses the intravasation of individual cells or CTC aggregates following tissue invasion, transit through the bloodstream, arrest, extravasation, and colonisation of target organ capillaries.^148^ These spatial events involve the timing of entry into circulation, microanatomical bottlenecks, and the environmental stimuli and selective pressures inherent in different target organs.^149^

**FIGURE 4:**
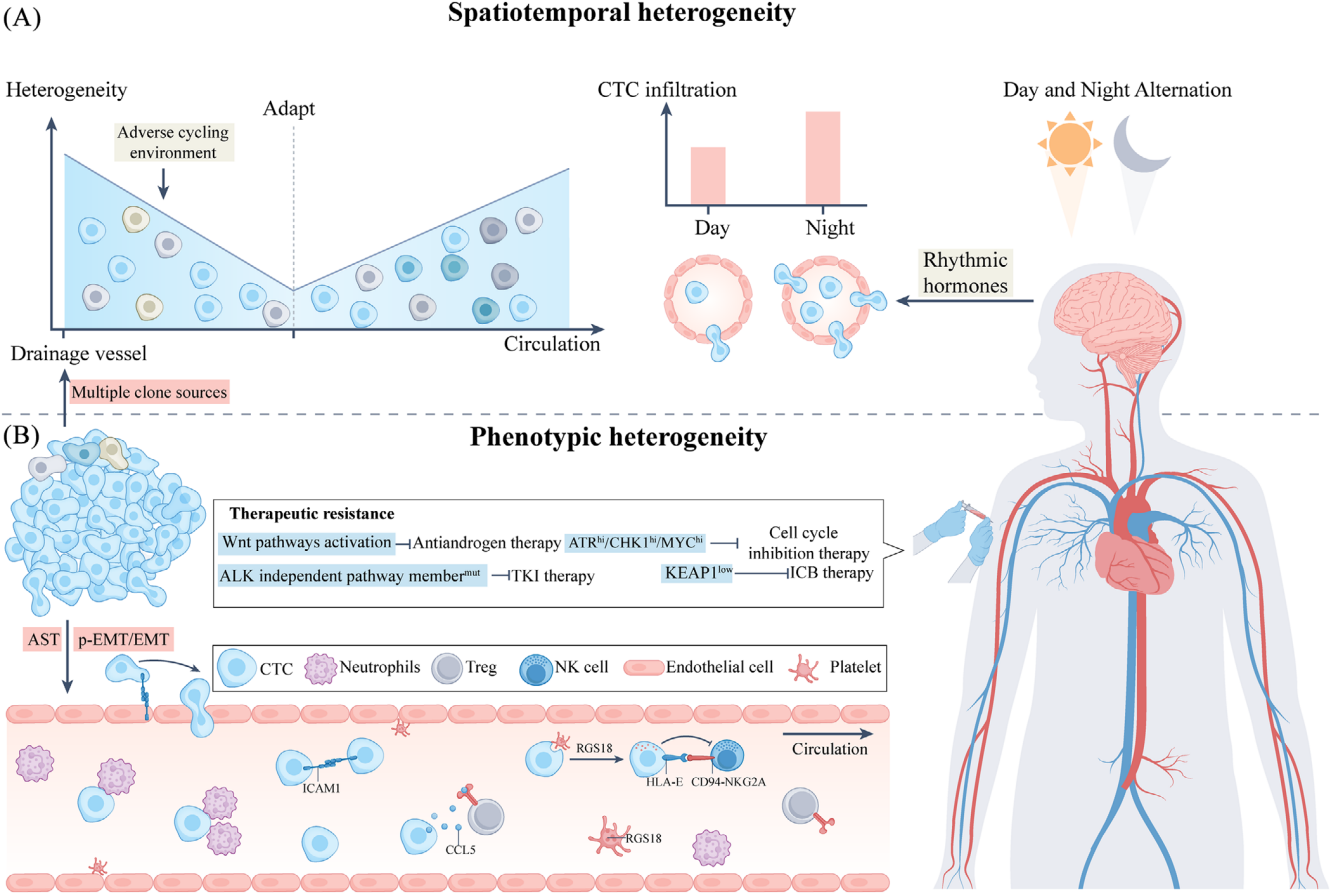
Spatiotemporal heterogeneity and phenotype of CTCs. (A) Polyclonally derived CTCs experience significant mortality upon initial entry into the challenging blood flow environment. As adaptation occurs, both viable and quiescent CTCs begin to proliferate, resulting in increased heterogeneity. Notably, CTC infiltration timing is influenced by the human body's circadian rhythm. During night‐time rest, intravasation of CTCs intensifies due to rhythmic hormonal effects. (B) Circulating CTCs exhibit phenotypic heterogeneity during their initial permeation. They undergo processes such as adherent‐suspension transition (AST), epithelial‐mesenchymal transition (EMT), and partial EMT (p‐EMT). Endothelial adhesion occurs via ICAM1‐ICAM1 homophilic interactions. Additionally, circulating ICAM1 molecules promote CTC cluster formation. Neutrophils also play a crucial role in CTC cluster formation. Functionally, circulating CTCs engage in immune evasion strategies to enhance their survival. These include recruiting Tregs through CCL5 secretion, phagocytosing platelet‐derived RGS18 to boost their own HLA‐E expression, and evading natural killer (NK)‐mediated immune surveillance by binding to the immune checkpoint HLA‐E: CD94‐NKG2A. Furthermore, CTCs may develop resistance mechanisms during treatment.

Recent studies suggest that CTC heterogeneity primarily manifests in two dimensions: the variability in afferent circulation and circadian rhythms (Figure 4A). As delineated in Chapter 2, primary tumours exhibit a variety of clonal mixtures, from which CTCs derive.^150^ Hence, CTCs of varying states can be detected within anatomically draining blood vessels across different regions of the primary tumour. For instance, Sun et al. applied scRNA‐seq to identify significant heterogeneity in the hepatic vein CTC population of hepatocellular carcinoma patients. And significant differences in CTC transcriptional heterogeneity were found in four vascular sites (hepatic vein, peripheral artery, peripheral vein and portal vein).^151^ This suggests that unfavorable circulatory conditions such as immune‐mediated selective pressure, anoikis, and physical shear stress contribute to molecular heterogeneity and phenotypic alterations in the circulatory system in which CTCs proceed.^152^, ^153^ Thus there is a dual heterogeneity arising from subpopulation origin and influence by the bloodstream environment. Recent scRNA‐seq analysis of circulating tumour cells (CTCs) collected from breast cancer patients during active (10:00 AM) and resting (4:00 AM) periods revealed significant upregulation of mitotic genes during the resting period, indicating that most spontaneous intravasation events of CTCs occur during sleep.^154^

Therefore, CTCs derived from polyclonal subpopulations undergo selection in response to changes in the bloodstream environment and circadian rhythms, leading to different biological characteristics at various anatomical sites and stages.^155^ It may explain the previously mentioned organotropism of metastasis and the diversity of monoclonal and mixed clonal landscapes in metastatic tumours. The next section will explore the biological phenotypes of CTCs in detail from a single‐cell sequencing perspective (Figure 4B).

### 5.2 Heterogeneity of phenotypes

When CTCs spread from the primary tumour and colonise distant sites they usually undergo EMT. Huh et al. observed that the occurrence of CTCs is associated with a phenomenon known as adhesion‐suspension transformation (AST), which orchestrates the reprogramming and shedding of in situ tumour cells into the bloodstream. Single‐cell sequencing revealed that key AST factors, including IKZF1, NFE2 and IRF8, were significantly enriched in CTCs.^156^ In another study, single‐cell RNA sequencing of mice with mammary tumours identified ICAM1 as a driver of breast cancer lung metastasis, and ICAM1‐ICAM1 homophilic interactions promoted the aggregation and adhesion of CTCs to tumour endothelial cells.^157^

CTCs exhibit the capacity for sustained division and proliferation within the bloodstream, with subsets displaying stem‐like properties.^158^ Cheng et al. identified ALDH overexpression in Mesenchymal‐epithelial transition‐like (MET‐like) CTCs, along with other established cancer stem cell (CSC) regulatory genes such as BMI1, GATA3, and SOX9.^159^ Consistent with recent findings suggesting that CTCs expressing epithelial and mesenchymal markers may have the highest potential for cellular plasticity and metastasis.^160^ Examination of 666 CTCs from 21 breast cancer patients using Hydro‐Seq with high CTC capture efficiency revealed elevated expression of cell cycle proteins in MET‐like CTCs, including D1 (CCND1) and c‐jun (JUN).^159^ Furthermore, the proliferative capacity of CTCs is related to the aggregates they form in the bloodstream. A study identified a neutrophil‐CTC ecotope effect in which CTCs aggregated with neutrophils showed higher proliferative potential than individual CTCs.^161^

CTCs evade immune surveillance through strategies such as secretion of immunosuppressive chemokines, interaction with platelets, and immune checkpoint‐mediated inhibition.^162^ Sun et al. identified CCL5 as a pivotal mediator in CTC immune evasion through scRNA‐seq. Specifically, CTCs recruit Tregs to foster CTC‐mediated metastasis by hyperactivating the p38‐MAX‐CCL5 signalling axis.^151^ Another study highlighted that CTCs evade immune surveillance by inhibiting NK cell‐mediated tumour cytotoxicity through HLA‐E and CD94‐NKG2A. Mechanistic research showed that platelet‐derived RGS18 enhances HLA‐E expression via the AKT‐GSK3β‐CREB signalling pathway, and this occurs through the HLA‐E immune checkpoint.^163^

### 5.3 Acquisition of drug resistance

Commonly used clinical cancer treatments include DNA cycle inhibitors, cell pathway inhibitors, and immunotherapy.^164^ scRNA‐seq analysis of xenograft models derived from small cell lung cancer CTCs revealed that recurrence following cisplatin treatment is associated with changes in EMT gene expression.^165^ Resistance to this drug was found to be associated with elevated ATR or CHK1 expression in single‐cell examinations of CTCs from patients with early‐stage breast cancer.^166^ Another single‐cell study found that in xenograft models of chemotherapy‐resistant non‐small cell lung cancer, there is an upregulation of MYC target genes and c‐MYC protein. Inhibition of MYC/MAX dimerisation may overcome cisplatin/paclitaxel resistance.^167^ Multiple mutations in genes within the ALK‐independent RTK‐KRAS and TP53 pathways, including EGFR, KRAS, and BRAF, have been identified at the single‐cell level in circulating tumour cells (CTCs) of crizotinib‐resistant patients.^168^ Another study indicates that non‐canonical Wnt signalling in prostate cancer is associated with anti‐androgen resistance.^169^ Fox et al. used SMART‐seq2 to find that the disease progression group of melanoma patients undergoing immune checkpoint blockade therapy had low expression of KEAP1 in CTCs, an effect mediated through the regulation of NRF2 activity.^170^ Single‐cell technologies have gradually been used to elucidate some mechanisms of drug resistance and identify potential targets.

## 6 CONCLUSIONS

**FIGURE 5:**
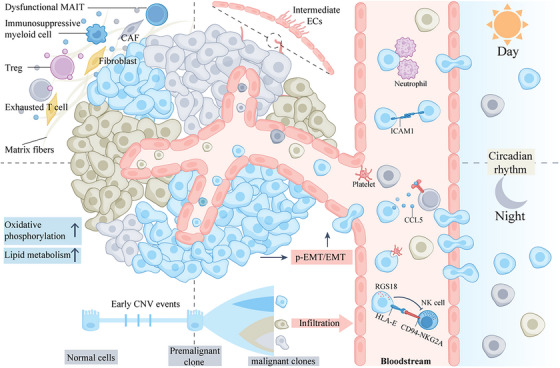
Graphical Abstract. Some tumours establish immunosuppressive and stromal barriers at their boundaries while enhancing oxidative phosphorylation and lipid metabolism to sustain their invasive potential. Additionally early evolving premalignant clones are able to cross histologic boundaries, possessing some genomic CNV features but not presenting cytomorphic changes. Multiple intratumoural clonal subpopulations infiltrate the blood vessels with a circadian rhythm when undergoing metastasis, while evading immunosurveillance and proliferating in the bloodstream in multiple ways.

Emerging single‐cell and spatial omics fields are currently elucidating the complex biological characteristics of tumours (Figure 5). Subclonal distributions have exhibited both inter‐cluster heterogeneity and intra‐cluster transcriptional regulatory diversity. Additionally, certain premalignant clones can cross tumour boundaries without cellular morphological changes. These insights underscore the value of genomic tissue analysis in enhancing prognostic and therapeutic evaluations within conventional clinical protocols

Characteristics at the tumour boundary are further elucidated by current cutting‐edge omics research. Dysfunctional T cell subgroups (characterised by T cell exhaustion), immunosuppressive myeloid subgroups marked by SPP1, PDCD1, or TERM2, and fibroblasts have been observed at the tumour boundary. This suggests that future research could focus on targeting these cellular subgroups at the boundary to activate immune responses and inhibit invasion. The invasive potential at the boundary can be partly characterised by angiogenesis, lipid metabolism, glucose metabolism, and multi‐stage EMT, making these factors potential targets for controlling tumour invasion in future local treatments considering spatial effects.

Shifting focus to haematogenous metastasis, the spatiotemporal heterogeneity of CTCs is a prominent distinction from solid primary tumour cells. CTCs collected from different vascular locations and at different times may exhibit potential biological characteristic differences. Therefore, when reviewing and comparing similar studies, spatiotemporal heterogeneity factors should be considered. Moreover, for clinical applications, appropriate standards for the timing and location should be established for CTC capture and biological characteristic detection.

## AUTHOR CONTRIBUTIONS

Qiongfang Yu, Dian Gao: conceptualisation, methodology, funding acquisition, supervision. Xifu Cheng, Yuke cao, Xiangyi Liu and Yuanheng Li: writing – original draft preparation and reviewing. Xifu Cheng and Yuke Cao: designed the figures. All the authors have read and approved the final version of the manuscript and agree with the order of presentation of the authors.

## CONFLICT OF INTEREST STATEMENT

The authors declare that they have no competing interests.

## ETHICS STATEMENT

These issues are not applicable for this review.

## CONSENT FOR PUBLICATION

The authors give their consent for the publication of the manuscript in *Clinical and Translational Medicine*.

## References

[1] Fujii M , Sekine S , Sato T . Decoding the basis of histological variation in human cancer. Nat Rev Cancer. 2024;24:141‐158. 38135758 10.1038/s41568-023-00648-5

[2] Koch C , Kuske A , Joosse SA , et al. Characterization of circulating breast cancer cells with tumorigenic and metastatic capacity. EMBO Mol Med. 2020;12:e11908. 32667137 10.15252/emmm.201911908 PMC7507517

[3] Zhang L , Ridgway LD , Wetzel MD , et al. The identification and characterization of breast cancer CTCs competent for brain metastasis. Sci Transl Med. 2013;5:180ra148. 10.1126/scitranslmed.3005109 PMC3863909 23576814

[4] Teltsh O , Porgador A , Rubin E . Extracting tumor tissue immune status from expression profiles: correlating renal cancer prognosis with tumor‐associated immunome. Oncotarget. 2015;6:33191‐33205. 26384298 10.18632/oncotarget.5052 PMC4741758

[5] Saunus JM , Quinn MC , Patch AM , et al. Integrated genomic and transcriptomic analysis of human brain metastases identifies alterations of potential clinical significance. J Pathol. 2015;237:363‐378. 26172396 10.1002/path.4583

[6] Richards S . It's more than stamp collecting: how genome sequencing can unify biological research. Trends Genet. 2015;31:411‐421. 26003218 10.1016/j.tig.2015.04.007 PMC4490122

[7] Thalgott M , Heck MM , Eiber M , et al. Circulating tumor cells versus objective response assessment predicting survival in metastatic castration‐resistant prostate cancer patients treated with docetaxel chemotherapy. J Cancer Res Clin Oncol. 2015;141:1457‐1464. 25708944 10.1007/s00432-015-1936-z PMC11824023

[8] Van der Auwera I , Peeters D , Benoy IH , et al. Circulating tumour cell detection: a direct comparison between the CellSearch System, the AdnaTest and CK‐19/mammaglobin RT‐PCR in patients with metastatic breast cancer. Br J Cancer. 2010;102:276‐284. 19953098 10.1038/sj.bjc.6605472 PMC2816650

[9] Vandereyken K , Sifrim A , Thienpont B , Voet T . Methods and applications for single‐cell and spatial multi‐omics. Nat Rev Genet. 2023;24:494‐515. 36864178 10.1038/s41576-023-00580-2 PMC9979144

[10] Casasent AK , Schalck A , Gao R , et al. Multiclonal invasion in breast tumors identified by topographic single cell sequencing. Cell. 2018;172:205‐217. e212. 29307488 10.1016/j.cell.2017.12.007 PMC5766405

[11] Zhao T , Chiang ZD , Morriss JW , et al. Spatial genomics enables multi‐modal study of clonal heterogeneity in tissues. Nature. 2022;601:85‐91. 34912115 10.1038/s41586-021-04217-4 PMC9301586

[12] Chen JH , Nieman LT , Spurrell M , et al. Human lung cancer harbors spatially organized stem‐immunity hubs associated with response to immunotherapy. Nat Immunol. 2024;25:644‐658. 38503922 10.1038/s41590-024-01792-2 PMC12096941

[13] Eng CL , Lawson M , Zhu Q , et al. Transcriptome‐scale super‐resolved imaging in tissues by RNA seqFISH. Nature. 2019;568:235‐239. 30911168 10.1038/s41586-019-1049-y PMC6544023

[14] Magen A , Hamon P , Fiaschi N , et al. Intratumoral dendritic cell‐CD4(+) T helper cell niches enable CD8(+) T cell differentiation following PD‐1 blockade in hepatocellular carcinoma. Nat Med. 2023;29:1389‐1399. 37322116 10.1038/s41591-023-02345-0 PMC11027932

[15] Lee JH , Daugharthy ER , Scheiman J , et al. Fluorescent in situ sequencing (FISSEQ) of RNA for gene expression profiling in intact cells and tissues. Nat Protoc. 2015;10:442‐458. 25675209 10.1038/nprot.2014.191 PMC4327781

[16] Lomakin A , Svedlund J , Strell C , et al. Spatial genomics maps the structure, nature and evolution of cancer clones. Nature. 2022;611:594‐602. 36352222 10.1038/s41586-022-05425-2 PMC9668746

[17] Rodriques SG , Stickels RR , Goeva A , et al. Slide‐seq: a scalable technology for measuring genome‐wide expression at high spatial resolution. Science. 2019;363:1463‐1467. 30923225 10.1126/science.aaw1219 PMC6927209

[18] Madissoon E , Oliver AJ , Kleshchevnikov V , et al. A spatially resolved atlas of the human lung characterizes a gland‐associated immune niche. Nat Genet. 2023;55:66‐77. 36543915 10.1038/s41588-022-01243-4 PMC9839452

[19] Oliveira MF , Romero JP , Chung M , et al. Characterization of immune cell populations in the tumor microenvironment of colorectal cancer using high definition spatial profiling. bioRxiv 2024:2024.2006.2004.597233.

[20] Janesick A , Shelansky R , Gottscho AD , et al. High resolution mapping of the tumor microenvironment using integrated single‐cell, spatial and in situ analysis. Nat Commun. 2023;14:8353. 38114474 10.1038/s41467-023-43458-x PMC10730913

[21] Galeano Nino JL , Wu H , LaCourse KD , et al. Effect of the intratumoral microbiota on spatial and cellular heterogeneity in cancer. Nature. 2022;611:810‐817. 36385528 10.1038/s41586-022-05435-0 PMC9684076

[22] Karras P , Bordeu I , Pozniak J , et al. A cellular hierarchy in melanoma uncouples growth and metastasis. Nature. 2022;610:190‐198. 36131018 10.1038/s41586-022-05242-7 PMC10439739

[23] Giesen C , Wang HA , Schapiro D , et al. Highly multiplexed imaging of tumor tissues with subcellular resolution by mass cytometry. Nat Methods. 2014;11:417‐422. 24584193 10.1038/nmeth.2869

[24] Keren L , Bosse M , Thompson S , et al. MIBI‐TOF: a multiplexed imaging platform relates cellular phenotypes and tissue structure. Sci Adv. 2019;5:eaax5851. 31633026 10.1126/sciadv.aax5851 PMC6785247

[25] Gerdes MJ , Sevinsky CJ , Sood A , et al. Highly multiplexed single‐cell analysis of formalin‐fixed, paraffin‐embedded cancer tissue. Proc Natl Acad Sci U S A. 2013;110:11982‐11987. 23818604 10.1073/pnas.1300136110 PMC3718135

[26] Du Z , Lin JR , Rashid R , et al. Qualifying antibodies for image‐based immune profiling and multiplexed tissue imaging. Nat Protoc. 2019;14:2900‐2930. 31534232 10.1038/s41596-019-0206-y PMC6959005

[27] Gut G , Herrmann MD , Pelkmans L . Multiplexed protein maps link subcellular organization to cellular states. Science. 2018;361:eaar7042. 30072512 10.1126/science.aar7042

[28] Schubert W , Bonnekoh B , Pommer AJ , et al. Analyzing proteome topology and function by automated multidimensional fluorescence microscopy. Nat Biotechnol. 2006;24:1270‐1278. 17013374 10.1038/nbt1250

[29] van den Brand M , Hoevenaars BM , et al. Sequential immunohistochemistry: a promising new tool for the pathology laboratory. Histopathology. 2014;65:651‐657. 24766252 10.1111/his.12446

[30] Schurch CM , Bhate SS , Barlow GL , et al. Coordinated cellular neighborhoods orchestrate antitumoral immunity at the colorectal cancer invasive front. Cell. 2020;182:1341‐1359. e1319. 32763154 10.1016/j.cell.2020.07.005 PMC7479520

[31] Saka SK , Wang Y , Kishi JY , et al. Immuno‐SABER enables highly multiplexed and amplified protein imaging in tissues. Nat Biotechnol. 2019;37:1080‐1090. 31427819 10.1038/s41587-019-0207-y PMC6728175

[32] Prentice BM , McMillen JC , Caprioli RM . Multiple TOF/TOF events in a single laser shot for multiplexed lipid identifications in MALDI imaging mass spectrometry. Int J Mass Spectrom. 2019;437:30‐37. 30906202 10.1016/j.ijms.2018.06.006 PMC6424509

[33] Wiseman JM , Ifa DR , Zhu Y , et al. Desorption electrospray ionization mass spectrometry: imaging drugs and metabolites in tissues. Proc Natl Acad Sci U S A. 2008;105:18120‐18125. 18697929 10.1073/pnas.0801066105 PMC2587601

[34] He J , Sun C , Li T , et al. A sensitive and wide coverage ambient mass spectrometry imaging method for functional metabolites based molecular histology. Adv Sci (Weinh). 2018;5:1800250. 30479912 10.1002/advs.201800250 PMC6247026

[35] Boxer SG , Kraft ML , Weber PK . Advances in imaging secondary ion mass spectrometry for biological samples. Annu Rev Biophys. 2009;38:53‐74. 19086820 10.1146/annurev.biophys.050708.133634

[36] Hou Y , Guo H , Cao C , et al. Single‐cell triple omics sequencing reveals genetic, epigenetic, and transcriptomic heterogeneity in hepatocellular carcinomas. Cell Res. 2016;26:304‐319. 26902283 10.1038/cr.2016.23 PMC4783472

[37] Schwartz GW , Zhou Y , Petrovic J , et al. TooManyCells identifies and visualizes relationships of single‐cell clades. Nat Methods. 2020;17:405‐413. 32123397 10.1038/s41592-020-0748-5 PMC7439807

[38] Cao J , Spielmann M , Qiu X , et al. The single‐cell transcriptional landscape of mammalian organogenesis. Nature. 2019;566:496‐502. 30787437 10.1038/s41586-019-0969-x PMC6434952

[39] Jin S , Guerrero‐Juarez CF , Zhang L , et al. Inference and analysis of cell‐cell communication using CellChat. Nat Commun. 2021;12:1088. 33597522 10.1038/s41467-021-21246-9 PMC7889871

[40] Nichterwitz S , Chen G , Aguila Benitez J , et al. Laser capture microscopy coupled with Smart‐seq2 for precise spatial transcriptomic profiling. Nat Commun. 2016;7:12139. 27387371 10.1038/ncomms12139 PMC4941116

[41] Stahl PL , Salmen F , Vickovic S , et al. Visualization and analysis of gene expression in tissue sections by spatial transcriptomics. Science. 2016;353:78‐82. 27365449 10.1126/science.aaf2403

[42] Dhainaut M , Rose SA , Akturk G , et al. Spatial CRISPR genomics identifies regulators of the tumor microenvironment. Cell. 2022;185:1223‐1239. e1220. 35290801 10.1016/j.cell.2022.02.015 PMC8992964

[43] Ravi VM , Will P , Kueckelhaus J , et al. Spatially resolved multi‐omics deciphers bidirectional tumor‐host interdependence in glioblastoma. Cancer Cell. 2022;40:639‐655. e613. 35700707 10.1016/j.ccell.2022.05.009

[44] Erickson A , He M , Berglund E , et al. Spatially resolved clonal copy number alterations in benign and malignant tissue. Nature. 2022;608:360‐367. 35948708 10.1038/s41586-022-05023-2 PMC9365699

[45] Tarabichi M , Salcedo A , Deshwar AG , et al. A practical guide to cancer subclonal reconstruction from DNA sequencing. Nat Methods. 2021;18:144‐155. 33398189 10.1038/s41592-020-01013-2 PMC7867630

[46] Fu X , Zhao Y , Lopez JI , et al. Spatial patterns of tumour growth impact clonal diversification in a computational model and the TRACERx Renal study. Nat Ecol Evol. 2022;6:88‐102. 34949820 10.1038/s41559-021-01586-x PMC8752443

[47] Elhanani O , Ben‐Uri R , Keren L . Spatial profiling technologies illuminate the tumor microenvironment. Cancer Cell. 2023;41:404‐420. 36800999 10.1016/j.ccell.2023.01.010

[48] Zahir N , Sun R , Gallahan D , Gatenby RA , Curtis C . Characterizing the ecological and evolutionary dynamics of cancer. Nat Genet. 2020;52:759‐767. 32719518 10.1038/s41588-020-0668-4

[49] Seferbekova Z , Lomakin A , Yates LR , Gerstung M . Spatial biology of cancer evolution. Nat Rev Genet. 2023;24:295‐313. 36494509 10.1038/s41576-022-00553-x

[50] Noble R , Burri D , Le Sueur C , et al. Spatial structure governs the mode of tumour evolution. Nat Ecol Evol. 2022;6:207‐217. 34949822 10.1038/s41559-021-01615-9 PMC8825284

[51] Jamal‐Hanjani M , Wilson GA , McGranahan N , et al. Tracking the evolution of non‐small‐cell lung cancer. N Engl J Med. 2017;376:2109‐2121. 28445112 10.1056/NEJMoa1616288

[52] Sun Y , Wu P , Zhang Z , et al. Integrated multi‐omics profiling to dissect the spatiotemporal evolution of metastatic hepatocellular carcinoma. Cancer Cell. 2024;42:135‐156. e117. 38101410 10.1016/j.ccell.2023.11.010

[53] Nguyen B , Fong C , Luthra A , et al. Genomic characterization of metastatic patterns from prospective clinical sequencing of 25,000 patients. Cell. 2022;185:563‐575. e511. 35120664 10.1016/j.cell.2022.01.003 PMC9147702

[54] Martinez‐Jimenez F , Movasati A , Brunner SR , et al. Pan‐cancer whole‐genome comparison of primary and metastatic solid tumours. Nature. 2023;618:333‐341. 37165194 10.1038/s41586-023-06054-z PMC10247378

[55] Gerlinger M , Rowan AJ , Horswell S , et al. Intratumor heterogeneity and branched evolution revealed by multiregion sequencing. N Engl J Med. 2012;366:883‐892. 22397650 10.1056/NEJMoa1113205 PMC4878653

[56] Liu X , Zhang K , Kaya NA , et al. Tumor phylogeography reveals block‐shaped spatial heterogeneity and the mode of evolution in hepatocellular carcinoma. Nat Commun. 2024;15:3169. 38609353 10.1038/s41467-024-47541-9 PMC11015015

[57] Ge G , Han Y , Zhang J , et al. Single‐cell RNA‐seq reveals a developmental hierarchy super‐imposed over subclonal evolution in the cellular ecosystem of prostate cancer. Adv Sci (Weinh). 2022;9:e2105530. 35322584 10.1002/advs.202105530 PMC9131431

[58] Coudray N , Ocampo PS , Sakellaropoulos T , et al. Classification and mutation prediction from non‐small cell lung cancer histopathology images using deep learning. Nat Med. 2018;24:1559‐1567. 30224757 10.1038/s41591-018-0177-5 PMC9847512

[59] Loeffler CML , Ortiz Bruechle N , Jung M , et al. Artificial intelligence‐based detection of FGFR3 mutational status directly from routine histology in bladder cancer: a possible preselection for molecular testing? Eur Urol Focus. 2022;8:472‐479. 33895087 10.1016/j.euf.2021.04.007

[60] Sundar R , Liu DH , Hutchins GG , et al. Spatial profiling of gastric cancer patient‐matched primary and locoregional metastases reveals principles of tumour dissemination. Gut. 2021;70:1823‐1832. 33229445 10.1136/gutjnl-2020-320805 PMC8458060

[61] Arthur DW , Winter KA , Kuerer HM , et al. Effectiveness of breast‐conserving surgery and 3‐dimensional conformal partial breast reirradiation for recurrence of breast cancer in the ipsilateral breast: the NRG Oncology/RTOG 1014 Phase 2 clinical trial. JAMA Oncol. 2020;6:75‐82. 31750868 10.1001/jamaoncol.2019.4320 PMC6902101

[62] Vicini FA , Cecchini RS , White JR , et al. Long‐term primary results of accelerated partial breast irradiation after breast‐conserving surgery for early‐stage breast cancer: a randomised, phase 3, equivalence trial. Lancet. 2019;394:2155‐2164. 31813636 10.1016/S0140-6736(19)32514-0 PMC7199428

[63] Deryugina EI , Kiosses WB . Intratumoral cancer cell intravasation can occur independent of invasion into the adjacent stroma. Cell Rep. 2017;19:601‐616. 28423322 10.1016/j.celrep.2017.03.064 PMC5659755

[64] Zlobec I , Lugli A . Invasive front of colorectal cancer: dynamic interface of pro‐/anti‐tumor factors. World J Gastroenterol. 2009;15:5898‐5906. 20014453 10.3748/wjg.15.5898 PMC2795176

[65] Sun X , Wu B , Chiang HC , et al. Tumour DDR1 promotes collagen fibre alignment to instigate immune exclusion. Nature. 2021;599:673‐678. 34732895 10.1038/s41586-021-04057-2 PMC8839149

[66] Davidson S , Efremova M , Riedel A , et al. Single‐cell RNA sequencing reveals a dynamic stromal niche that supports tumor growth. Cell Rep. 2020;31:107628. 32433953 10.1016/j.celrep.2020.107628 PMC7242909

[67] Wu F , Zhang X , Wang M , et al. Deciphering the role of immunoglobulin secreting malignant lineages in the invasive frontiers of small cell lung cancer by scRNA‐seq and spatial transcriptomics analysis. Cell Discov. 2023;9:123. 38081849 10.1038/s41421-023-00621-4 PMC10713609

[68] Nirmal AJ , Maliga Z , Vallius T , et al. The spatial landscape of progression and immunoediting in primary melanoma at single‐cell resolution. Cancer Discov. 2022;12:1518‐1541. 35404441 10.1158/2159-8290.CD-21-1357 PMC9167783

[69] Tora MS , Neill SG , Lakhina Y , et al. Tumor microenvironment in a minipig model of spinal cord glioma. J Transl Med. 2023;21:667. 37752585 10.1186/s12967-023-04531-7 PMC10523785

[70] Davis S , Scott C , Oetjen J , et al. Deep topographic proteomics of a human brain tumour. Nat Commun. 2023;14:7710. 38001067 10.1038/s41467-023-43520-8 PMC10673928

[71] Xun Z , Ding X , Zhang Y , et al. Reconstruction of the tumor spatial microenvironment along the malignant‐boundary‐nonmalignant axis. Nat Commun. 2023;14:933. 36806082 10.1038/s41467-023-36560-7 PMC9941488

[72] Danenberg E , Bardwell H , Zanotelli VRT , et al. Breast tumor microenvironment structures are associated with genomic features and clinical outcome. Nat Genet. 2022;54:660‐669. 35437329 10.1038/s41588-022-01041-y PMC7612730

[73] Du Y , Shi J , Wang J , et al. Integration of pan‐cancer single‐cell and spatial transcriptomics reveals stromal cell features and therapeutic targets in tumor microenvironment. Cancer Res. 2024;84:192‐210. 38225927 10.1158/0008-5472.CAN-23-1418

[74] Liu Y , Xun Z , Ma K , et al. Identification of a tumour immune barrier in the HCC microenvironment that determines the efficacy of immunotherapy. J Hepatol. 2023;78:770‐782. 36708811 10.1016/j.jhep.2023.01.011

[75] Wu R , Guo W , Qiu X , et al. Comprehensive analysis of spatial architecture in primary liver cancer. Sci Adv. 2021;7:eabg3750. 34919432 10.1126/sciadv.abg3750 PMC8683021

[76] Fan G , Xie T , Li L , Tang L , Han X , Shi Y . Single‐cell and spatial analyses revealed the co‐location of cancer stem cells and SPP1+ macrophage in hypoxic region that determines the poor prognosis in hepatocellular carcinoma. NPJ Precis Oncol. 2024;8:75. 38521868 10.1038/s41698-024-00564-3 PMC10960828

[77] Schmassmann P , Roux J , Dettling S , et al. Single‐cell characterization of human GBM reveals regional differences in tumor‐infiltrating leukocyte activation. Elife. 2023;12:RP92678. 38127790 10.7554/eLife.92678 PMC10735226

[78] Chen Z , Soifer I , Hilton H , Keren L , Jojic V . Modeling multiplexed images with spatial‐LDA reveals novel tissue microenvironments. J Comput Biol. 2020;27:1204‐1218. 32243203 10.1089/cmb.2019.0340 PMC7415889

[79] He S , Jin Y , Nazaret A , et al. Starfysh integrates spatial transcriptomic and histologic data to reveal heterogeneous tumor‐immune hubs. Nat Biotechnol. 2024. 10.1038/s41587-024-02173-8 PMC11415552 38514799

[80] Ruf B , Bruhns M , Babaei S , et al. Tumor‐associated macrophages trigger MAIT cell dysfunction at the HCC invasive margin. Cell. 2023;186:3686‐3705. e3632. 37595566 10.1016/j.cell.2023.07.026 PMC10461130

[81] Zheng B , Wang D , Qiu X , et al. Trajectory and functional analysis of PD‐1(high) CD4(+)CD8(+) T cells in hepatocellular carcinoma by single‐cell cytometry and transcriptome sequencing. Adv Sci (Weinh). 2020;7:2000224. 32670760 10.1002/advs.202000224 PMC7341083

[82] Qi J , Sun H , Zhang Y , et al. Single‐cell and spatial analysis reveal interaction of FAP(+) fibroblasts and SPP1(+) macrophages in colorectal cancer. Nat Commun. 2022;13:1742. 35365629 10.1038/s41467-022-29366-6 PMC8976074

[83] Ozato Y , Kojima Y , Kobayashi Y , et al. Spatial and single‐cell transcriptomics decipher the cellular environment containing HLA‐G+ cancer cells and SPP1+ macrophages in colorectal cancer. Cell Rep. 2023;42:111929. 36656712 10.1016/j.celrep.2022.111929

[84] Wu L , Yan J , Bai Y , et al. An invasive zone in human liver cancer identified by Stereo‐seq promotes hepatocyte‐tumor cell crosstalk, local immunosuppression and tumor progression. Cell Res. 2023;33:585‐603. 37337030 10.1038/s41422-023-00831-1 PMC10397313

[85] Yofe I , Shami T , Cohen N , et al. Spatial and temporal mapping of breast cancer lung metastases identify TREM2 macrophages as regulators of the metastatic boundary. Cancer Discov. 2023;13:2610‐2631. 37756565 10.1158/2159-8290.CD-23-0299 PMC7617931

[86] Altunbulakli C , Jimenez DG , Askmyr D , et al. Targeted spatial proteomic analysis of CD8(+) T‐ and myeloid cells in tonsillar cancer. Front Oncol. 2023;13:1253418. 38044986 10.3389/fonc.2023.1253418 PMC10691541

[87] Yan X , Xie Y , Yang F , et al. Comprehensive description of the current breast cancer microenvironment advancements via single‐cell analysis. J Exp Clin Cancer Res. 2021;40:142. 33906694 10.1186/s13046-021-01949-z PMC8077685

[88] Hu H , Piotrowska Z , Hare PJ , et al. Three subtypes of lung cancer fibroblasts define distinct therapeutic paradigms. Cancer Cell. 2021;39:1531‐1547. e1510. 34624218 10.1016/j.ccell.2021.09.003 PMC8578451

[89] Chhabra Y , Weeraratna AT . Fibroblasts in cancer: unity in heterogeneity. Cell. 2023;186:1580‐1609. 37059066 10.1016/j.cell.2023.03.016 PMC11422789

[90] Baessler A , Vignali DAA . T cell exhaustion. Annu Rev Immunol. 2024;42(1):179‐206. 38166256 10.1146/annurev-immunol-090222-110914

[91] Miggelbrink AM , Jackson JD , Lorrey SJ , et al. CD4 T‐cell exhaustion: does it exist and what are its roles in cancer? Clin Cancer Res. 2021;27:5742‐5752. 34127507 10.1158/1078-0432.CCR-21-0206 PMC8563372

[92] Liu C , Lan Y , Liu B , Zhang H , Hu H . T cell development: old tales retold by single‐cell RNA sequencing. Trends Immunol. 2021;42:165‐175. 33446417 10.1016/j.it.2020.12.004

[93] Cunha MT , Gouveia MC , Neto FL , et al. Long‐term outcomes of neoadjuvant immunotherapy plus chemotherapy in patients with early‐stage triple‐negative breast cancer: an extracted individual patient data and trial‐level meta‐analysis. Br J Cancer. 2024;130:242‐250. 38012381 10.1038/s41416-023-02501-w PMC10803354

[94] Blanco‐Heredia J , Souza CA , Trincado JL , et al. Converging and evolving immuno‐genomic routes toward immune escape in breast cancer. Nat Commun. 2024;15:1302. 38383522 10.1038/s41467-024-45292-1 PMC10882008

[95] Barras D , Ghisoni E , Chiffelle J , et al. Response to tumor‐infiltrating lymphocyte adoptive therapy is associated with preexisting CD8(+) T‐myeloid cell networks in melanoma. Sci Immunol. 2024;9:eadg7995. 38306416 10.1126/sciimmunol.adg7995

[96] Gabriely G , Ma D , Siddiqui S , et al. Myeloid cell subsets that express latency‐associated peptide promote cancer growth by modulating T cells. iScience. 2021;24:103347. 34820606 10.1016/j.isci.2021.103347 PMC8602030

[97] Cheng S , Li Z , Gao R , et al. A pan‐cancer single‐cell transcriptional atlas of tumor infiltrating myeloid cells. Cell. 2021;184:792‐809. e723. 33545035 10.1016/j.cell.2021.01.010

[98] Barkley D , Moncada R , Pour M , et al. Cancer cell states recur across tumor types and form specific interactions with the tumor microenvironment. Nat Genet. 2022;54:1192‐1201. 35931863 10.1038/s41588-022-01141-9 PMC9886402

[99] Zhang L , Li Z , Skrzypczynska KM , et al. Single‐cell analyses inform mechanisms of myeloid‐targeted therapies in colon cancer. Cell. 2020;181:442‐459. e429. 32302573 10.1016/j.cell.2020.03.048

[100] Liu Y , Zhang Q , Xing B , et al. Immune phenotypic linkage between colorectal cancer and liver metastasis. Cancer Cell. 2022;40:424‐437. e425. 35303421 10.1016/j.ccell.2022.02.013

[101] Mantovani A , Allavena P , Marchesi F , Garlanda C . Macrophages as tools and targets in cancer therapy. Nat Rev Drug Discov. 2022;21:799‐820. 35974096 10.1038/s41573-022-00520-5 PMC9380983

[102] Molgora M , Liu YA , Colonna M , Cella M . TREM2: a new player in the tumor microenvironment. Semin Immunol. 2023;67:101739. 36989543 10.1016/j.smim.2023.101739

[103] Ji KY , Kim SM , Yee SM , et al. Cyclophilin A is an endogenous ligand for the triggering receptor expressed on myeloid cells‐2 (TREM2). FASEB J. 2021;35:e21479. 33710680 10.1096/fj.202002325RR

[104] Binnewies M , Pollack JL , Rudolph J , et al. Targeting TREM2 on tumor‐associated macrophages enhances immunotherapy. Cell Rep. 2021;37:109844. 34686340 10.1016/j.celrep.2021.109844

[105] Zhang Y , Chen H , Mo H , et al. Single‐cell analyses reveal key immune cell subsets associated with response to PD‐L1 blockade in triple‐negative breast cancer. Cancer Cell. 2021;39:1578‐1593. e1578. 34653365 10.1016/j.ccell.2021.09.010

[106] Molgora M , Esaulova E , Vermi W , et al. TREM2 Modulation remodels the tumor myeloid landscape enhancing anti‐PD‐1 immunotherapy. Cell. 2020;182:886‐900. e817. 32783918 10.1016/j.cell.2020.07.013 PMC7485282

[107] Shi X , Pang S , Zhou J , et al. Bladder‐cancer‐derived exosomal circRNA_0013936 promotes suppressive immunity by up‐regulating fatty acid transporter protein 2 and down‐regulating receptor‐interacting protein kinase 3 in PMN‐MDSCs. Mol Cancer. 2024;23:52. 38461272 10.1186/s12943-024-01968-2 PMC10924381

[108] Long H , Jia Q , Wang L , et al. Tumor‐induced erythroid precursor‐differentiated myeloid cells mediate immunosuppression and curtail anti‐PD‐1/PD‐L1 treatment efficacy. Cancer Cell. 2022;40:674‐693. e677. 35594863 10.1016/j.ccell.2022.04.018

[109] Zhu L , Yu X , Wang L , et al. Angiogenesis and immune checkpoint dual blockade in combination with radiotherapy for treatment of solid cancers: opportunities and challenges. Oncogenesis. 2021;10:47. 34247198 10.1038/s41389-021-00335-w PMC8272720

[110] Geldhof V , de Rooij L , Sokol L , et al. Single cell atlas identifies lipid‐processing and immunomodulatory endothelial cells in healthy and malignant breast. Nat Commun. 2022;13:5511. 36127427 10.1038/s41467-022-33052-y PMC9489707

[111] Zhao Q , Molina‐Portela MDP , Parveen A , et al. Heterogeneity and chimerism of endothelial cells revealed by single‐cell transcriptome in orthotopic liver tumors. Angiogenesis. 2020;23:581‐597. 32440964 10.1007/s10456-020-09727-9 PMC7525283

[112] Goveia J , Rohlenova K , Taverna F , et al. An integrated gene expression landscape profiling approach to identify lung tumor endothelial cell heterogeneity and angiogenic candidates. Cancer Cell. 2020;37:21‐36. e13. 31935371 10.1016/j.ccell.2019.12.001

[113] Sharma A , Seow JJW , Dutertre CA , et al. Onco‐fetal reprogramming of endothelial cells drives immunosuppressive macrophages in hepatocellular carcinoma. Cell. 2020;183:377‐394. e321. 32976798 10.1016/j.cell.2020.08.040

[114] Zhou PY , Zhou C , Gan W , et al. Single‐cell and spatial architecture of primary liver cancer. Commun Biol. 2023;6:1181. 37985711 10.1038/s42003-023-05455-0 PMC10661180

[115] Zeng Q , Mousa M , Nadukkandy AS , et al. Understanding tumour endothelial cell heterogeneity and function from single‐cell omics. Nat Rev Cancer. 2023;23:544‐564. 37349410 10.1038/s41568-023-00591-5

[116] Huang P , Fan X , Yu H , et al. Glucose metabolic reprogramming and its therapeutic potential in obesity‐associated endometrial cancer. J Transl Med. 2023;21:94. 36750868 10.1186/s12967-022-03851-4 PMC9906873

[117] Mancini C , Lori G , Pranzini E , Taddei ML . Metabolic challengers selecting tumor‐persistent cells. Trends Endocrinol Metab. 2024;35:263‐276. 38071164 10.1016/j.tem.2023.11.005

[118] Sung JY , Cheong JH . Intercellular communications and metabolic reprogramming as new predictive markers for immunotherapy responses in gastric cancer. Cancer Commun (Lond). 2022;42:572‐575. 35396921 10.1002/cac2.12285 PMC9198349

[119] Alexandrov T . Spatial metabolomics: from a niche field towards a driver of innovation. Nat Metab. 2023;5:1443‐1445. 37679554 10.1038/s42255-023-00881-0

[120] Wang J , Kunzke T , Prade VM , et al. Spatial metabolomics identifies distinct tumor‐specific subtypes in gastric cancer patients. Clin Cancer Res. 2022;28:2865‐2877. 35395077 10.1158/1078-0432.CCR-21-4383

[121] Chen Z , Han F , Du Y , Shi H , Zhou W . Hypoxic microenvironment in cancer: molecular mechanisms and therapeutic interventions. Signal Transduct Target Ther. 2023;8:70. 36797231 10.1038/s41392-023-01332-8 PMC9935926

[122] Banerjee S , Zare RN , Tibshirani RJ , et al. Diagnosis of prostate cancer by desorption electrospray ionization mass spectrometric imaging of small metabolites and lipids. Proc Natl Acad Sci U S A. 2017;114:3334‐3339. 28292895 10.1073/pnas.1700677114 PMC5380053

[123] Vogel FCE , Chaves‐Filho AB , Schulze A . Lipids as mediators of cancer progression and metastasis. Nat Cancer. 2024;5:16‐29. 38273023 10.1038/s43018-023-00702-z

[124] Randall EC , Lopez BGC , Peng S , et al. Localized metabolomic gradients in patient‐derived xenograft models of glioblastoma. Cancer Res. 2020;80:1258‐1267. 31767628 10.1158/0008-5472.CAN-19-0638 PMC7073296

[125] Wang M , Deng C , Yang C , et al. Unraveling temporal and spatial biomarkers of epithelial‐mesenchymal transition in colorectal cancer: insights into the crucial role of immunosuppressive cells. J Transl Med. 2023;21:794. 37940972 10.1186/s12967-023-04600-x PMC10633927

[126] Liu YM , Ge JY , Chen YF , et al. Combined single‐cell and spatial transcriptomics reveal the metabolic evolvement of breast cancer during early dissemination. Adv Sci (Weinh). 2023;10:e2205395. 36594618 10.1002/advs.202205395 PMC9951304

[127] Fiore VF , Krajnc M , Quiroz FG , et al. Mechanics of a multilayer epithelium instruct tumour architecture and function. Nature. 2020;585:433‐439. 32879493 10.1038/s41586-020-2695-9 PMC7787055

[128] Du W , Nair P , Johnston A , Wu PH , Wirtz D . Cell trafficking at the intersection of the tumor‐immune compartments. Annu Rev Biomed Eng. 2022;24:275‐305. 35385679 10.1146/annurev-bioeng-110320-110749 PMC9811395

[129] Dongre A , Weinberg RA . New insights into the mechanisms of epithelial‐mesenchymal transition and implications for cancer. Nat Rev Mol Cell Biol. 2019;20:69‐84. 30459476 10.1038/s41580-018-0080-4

[130] Li R , Ferdinand JR , Loudon KW , et al. Mapping single‐cell transcriptomes in the intra‐tumoral and associated territories of kidney cancer. Cancer Cell. 2022;40:1583‐1599. e1510. 36423636 10.1016/j.ccell.2022.11.001 PMC9767677

[131] Cao J , Zheng Z , Sun D , et al. Decoder‐seq enhances mRNA capture efficiency in spatial RNA sequencing. Nat Biotechnol. 2024. 10.1038/s41587-023-02086-y 38228777

[132] Fujiyoshi K , Vayrynen JP , Borowsky J , et al. Tumour budding, poorly differentiated clusters, and T‐cell response in colorectal cancer. EBioMedicine. 2020;57:102860. 32652320 10.1016/j.ebiom.2020.102860 PMC7347996

[133] Wei L , Delin Z , Kefei Y , Hong W , Jiwei H , Yange Z . A classification based on tumor budding and immune score for patients with hepatocellular carcinoma. Oncoimmunology. 2020;9:1672495. 32002283 10.1080/2162402X.2019.1672495 PMC6959452

[134] Zuo L , Li W , You S . Progesterone reverses the mesenchymal phenotypes of basal phenotype breast cancer cells via a membrane progesterone receptor mediated pathway. Breast Cancer Res. 2010;12:R34. 20540763 10.1186/bcr2588 PMC2917029

[135] Zhu XL , Wang YL , Chen JP , et al. Alternol inhibits migration and invasion of human hepatocellular carcinoma cells by targeting epithelial‐to‐mesenchymal transition. Tumour Biol. 2014;35:1627‐1635. 24078466 10.1007/s13277-013-1224-y

[136] Zhu X , Zhong J , Zhao Z , et al. Epithelial derived CTGF promotes breast tumor progression via inducing EMT and collagen I fibers deposition. Oncotarget. 2015;6:25320‐25338. 26318291 10.18632/oncotarget.4659 PMC4694834

[137] Pastushenko I , Brisebarre A , Sifrim A , et al. Identification of the tumour transition states occurring during EMT. Nature. 2018;556:463‐468. 29670281 10.1038/s41586-018-0040-3

[138] Malagoli Tagliazucchi G , Wiecek AJ , Withnell E , Secrier M . Genomic and microenvironmental heterogeneity shaping epithelial‐to‐mesenchymal trajectories in cancer. Nat Commun. 2023;14:789. 36774358 10.1038/s41467-023-36439-7 PMC9922305

[139] Puram SV , Tirosh I , Parikh AS , et al. Single‐cell transcriptomic analysis of primary and metastatic tumor ecosystems in head and neck cancer. Cell. 2017;171:1611‐1624. e1624. 29198524 10.1016/j.cell.2017.10.044 PMC5878932

[140] Zhang Q , Fei L , Han R , et al. Single‐cell transcriptome reveals cellular hierarchies and guides p‐EMT‐targeted trial in skull base chordoma. Cell Discov. 2022;8:94. 36127333 10.1038/s41421-022-00459-2 PMC9489773

[141] Morikawa M , Derynck R , Miyazono K . TGF‐beta and the TGF‐beta family: context‐dependent roles in cell and tissue physiology. Cold Spring Harb Perspect Biol. 2016;8:a021873. 27141051 10.1101/cshperspect.a021873 PMC4852809

[142] Macias MJ , Martin‐Malpartida P , Massague J . Structural determinants of Smad function in TGF‐beta signaling. Trends Biochem Sci. 2015;40:296‐308. 25935112 10.1016/j.tibs.2015.03.012 PMC4485443

[143] Katsuno Y , Derynck R . Epithelial plasticity, epithelial‐mesenchymal transition, and the TGF‐beta family. Dev Cell. 2021;56:726‐746. 33756119 10.1016/j.devcel.2021.02.028

[144] Zoni E , van der Pluijm G , Gray PC , Kruithof‐de Julio M . Epithelial plasticity in cancer: unmasking a MicroRNA network for TGF‐beta‐, Notch‐, and Wnt‐mediated EMT. J Oncol. 2015;2015:198967. 25883651 10.1155/2015/198967 PMC4390187

[145] Ye Z , Li Q , Hu Y , et al. The stromal microenvironment endows pancreatic neuroendocrine tumors with spatially specific invasive and metastatic phenotypes. Cancer Lett. 2024;588:216769. 38438098 10.1016/j.canlet.2024.216769

[146] Lambert AW , Pattabiraman DR , Weinberg RA . Emerging biological principles of metastasis. Cell. 2017;168:670‐691. 28187288 10.1016/j.cell.2016.11.037 PMC5308465

[147] Csonti K , Fazakas C , Molnar K , Wilhelm I , Krizbai IA , Vegh AG . Breast adenocarcinoma cells adhere stronger to brain pericytes than to endothelial cells. Colloids Surf B Biointerfaces. 2024;234:113751. 38241889 10.1016/j.colsurfb.2024.113751

[148] Gerstberger S , Jiang Q , Ganesh K . Metastasis. Cell. 2023;186:1564‐1579. 37059065 10.1016/j.cell.2023.03.003 PMC10511214

[149] Lin D , Shen L , Luo M , et al. Circulating tumor cells: biology and clinical significance. Signal Transduct Target Ther. 2021;6:404. 34803167 10.1038/s41392-021-00817-8 PMC8606574

[150] Limaye S , Chowdhury S , Rohatgi N , et al. Accurate prostate cancer detection based on enrichment and characterization of prostate cancer specific circulating tumor cells. Cancer Med. 2023;12:9116‐9127. 36718027 10.1002/cam4.5649 PMC10166919

[151] Sun YF , Wu L , Liu SP , et al. Dissecting spatial heterogeneity and the immune‐evasion mechanism of CTCs by single‐cell RNA‐seq in hepatocellular carcinoma. Nat Commun. 2021;12:4091. 34215748 10.1038/s41467-021-24386-0 PMC8253833

[152] Sun YF , Guo W , Xu Y , et al. circulating tumor cells from different vascular sites exhibit spatial heterogeneity in epithelial and mesenchymal composition and distinct clinical significance in hepatocellular carcinoma. Clin Cancer Res. 2018;24:547‐559. 29070526 10.1158/1078-0432.CCR-17-1063

[153] Dong X , Ma Y , Zhao X , et al. Spatial heterogeneity in epithelial to mesenchymal transition properties of circulating tumor cells associated with distant recurrence in pancreatic cancer patients. Ann Transl Med. 2020;8:676. 32617296 10.21037/atm-20-782 PMC7327339

[154] Diamantopoulou Z , Castro‐Giner F , Schwab FD , et al. The metastatic spread of breast cancer accelerates during sleep. Nature. 2022;607:156‐162. 35732738 10.1038/s41586-022-04875-y

[155] Yu T , Wang C , Xie M , et al. Heterogeneity of CTC contributes to the organotropism of breast cancer. Biomed Pharmacother. 2021;137:111314. 33581649 10.1016/j.biopha.2021.111314

[156] Huh HD , Sub Y , Oh J , et al. Reprogramming anchorage dependency by adherent‐to‐suspension transition promotes metastatic dissemination. Mol Cancer. 2023;22:63. 36991428 10.1186/s12943-023-01753-7 PMC10061822

[157] Taftaf R , Liu X , Singh S , et al. ICAM1 initiates CTC cluster formation and trans‐endothelial migration in lung metastasis of breast cancer. Nat Commun. 2021;12:4867. 34381029 10.1038/s41467-021-25189-z PMC8358026

[158] Lah TT , Novak M , Breznik B . Brain malignancies: glioblastoma and brain metastases. Semin Cancer Biol. 2020;60:262‐273. 31654711 10.1016/j.semcancer.2019.10.010

[159] Cheng YH , Chen YC , Lin E , et al. Hydro‐Seq enables contamination‐free high‐throughput single‐cell RNA‐sequencing for circulating tumor cells. Nat Commun. 2019;10:2163. 31092822 10.1038/s41467-019-10122-2 PMC6520360

[160] Shaikh MV , Kala M , Nivsarkar M . CD90 a potential cancer stem cell marker and a therapeutic target. Cancer Biomark. 2016;16:301‐307. 27062695 10.3233/CBM-160590 PMC13016484

[161] Szczerba BM , Castro‐Giner F , Vetter M , et al. Neutrophils escort circulating tumour cells to enable cell cycle progression. Nature. 2019;566:553‐557. 30728496 10.1038/s41586-019-0915-y

[162] Rupp B , Ball H , Wuchu F , Nagrath D , Nagrath S . Circulating tumor cells in precision medicine: challenges and opportunities. Trends Pharmacol Sci. 2022;43:378‐391. 35272862 10.1016/j.tips.2022.02.005

[163] Liu X , Song J , Zhang H , et al. Immune checkpoint HLA‐E:cD94‐NKG2A mediates evasion of circulating tumor cells from NK cell surveillance. Cancer Cell. 2023;41:272‐287. e279. 36706761 10.1016/j.ccell.2023.01.001

[164] van de Haar J , Mankor JM , Hummelink K , et al. Combining genomic biomarkers to guide immunotherapy in non‐small cell lung cancer. Clin Cancer Res. 2024;30:1307‐1318. 38300729 10.1158/1078-0432.CCR-23-4027 PMC10982639

[165] Stewart CA , Gay CM , Xi Y , et al. Single‐cell analyses reveal increased intratumoral heterogeneity after the onset of therapy resistance in small‐cell lung cancer. Nat Cancer. 2020;1:423‐436. 33521652 10.1038/s43018-019-0020-z PMC7842382

[166] Pang S , Xu S , Wang L , et al. Molecular profiles of single circulating tumor cells from early breast cancer patients with different lymph node statuses. Thorac Cancer. 2023;14:156‐167. 36408679 10.1111/1759-7714.14728 PMC9834698

[167] Suvilesh KN , Nussbaum YI , Radhakrishnan V , et al. Tumorigenic circulating tumor cells from xenograft mouse models of non‐metastatic NSCLC patients reveal distinct single cell heterogeneity and drug responses. Mol Cancer. 2022;21:73. 35279152 10.1186/s12943-022-01553-5 PMC8917773

[168] Pailler E , Faugeroux V , Oulhen M , et al. Acquired resistance mutations to ALK inhibitors identified by single circulating tumor cell sequencing in ALK‐rearranged non‐small‐cell lung cancer. Clin Cancer Res. 2019;25:6671‐6682. 31439588 10.1158/1078-0432.CCR-19-1176

[169] Zhang Z , Xu Y . FZD7 accelerates hepatic metastases in pancreatic cancer by strengthening EMT and stemness associated with TGF‐beta/SMAD3 signaling. Mol Med. 2022;28:82. 35854234 10.1186/s10020-022-00509-1 PMC9295360

[170] Fox DB , Ebright RY , Hong X , et al. Downregulation of KEAP1 in melanoma promotes resistance to immune checkpoint blockade. NPJ Precis Oncol. 2023;7:25. 36864091 10.1038/s41698-023-00362-3 PMC9981575

